# Supplementary material for: A Prospective Analysis of the Burden of Multi-Drug-Resistant Pathogens in Acute Appendicitis and Their Implication for Clinical Management
Source: Antibiotics (Basel). 2025 Apr 4;14(4):378. doi: 10.3390/antibiotics14040378 (PMC12024413; doi:10.3390/antibiotics14040378)
Supplement: Supplementary file 1 [file antibiotics-14-00378-s001.zip › antibiotics-3536547-supplementary.pdf]

Supplementary Materials

Tables

Supp. Table 1: Detected microorganisms and corresponding antibiotic resistance profiles in the non-multi-drug resistant (non-MDR) group from appendiceal swab samples

**Microorganism**	**Resistant antibiotics**	**N**
No detection of miroorgansims		34
Escherichia coli	No resistance	10
Escherichia coli, Bacteroides spp.	No resistance	2
Cutibacterium acnes	No resistance	3
Streptococcus anginosus, Bacteroides fragilis/ovatus/xylanisolvens	No resistance	1
Escherichia coli, Enterococcus avium, Bacteroides fragilis	No resistance	1
Bacteriodes fragilis	No resistance	1
Staphylococcus epidermidis	No resistance	1
Pseudomonas aeruginosa, Escherichia coli, Streptococcus anginosus	No resistance	1
Escherichia coli, Citrobacter koseri	No resistance	1
Streptococcus anginosus, Fusobacterium nucleatum, Bacteroides, Eghertella lenta	No resistance	1
Escherichia coli, Clostridium innocuum	No resistance	1
Peptostreptococcus micros	No resistance	1
Streptococcus mitis, Streptococcus oralis	No resistance	1
Escherichia coli, Schaalia turicensis	No resistance	1
Pseudomonas aeruginosa	Fosfomycin	1
Escherichia coli, Bacteroides spp.	Ampicillin	1
Escherichia coli	Ampicillin/sulbactam	1
Escherichia coli	Ampicillin	1
Escherichia coli, Citrobacter amalonaticus	Ampicillin	1
Pseudomonas aeruginosa, Escherichia coli, Streptococcus constellatus, Enterococcus avium, Bacteroides distasonis	Fosfomycin	1
Enterococcus avium, Bacteroides fragilis	Clindamycin	1
Pseudomonas aeruginosa, Streptococcus constellatus, Bacteroides	Clindamycin	1
Klebsiella pneumoniae	Ampicillin	1
Escherichia coli	Amicillin/sulbactam	1
Staphylococcus lugdunensis, Bacteroides fragilis	Ampicillin	1
Escherichia coli, Enterococcus avium, Bacteroides ovatus	Ampicillin/sulbactam	1
Escherichia coli, Enterococcus faecalis, Candida albicans	Ciprofloxacin	1
Enterococcus faecium, Escherichia coli, Candida albicans	Ampicillin	1
Corynebacterium propinquum	Clindamycin	1
Escherichia coli, Enterococcus faecalis, Bacteroides distasonis, Bacteroides ovatus, Bacteroides xylanisolvens	Ampicillin/sulbactam	1

ESBL-R- Extended spectrum beta-lactamase resistance, 3MRGN- Gram-negative rods that are multi-drug resistant to three of four classes of antibiotics, non-MDR – non-multi-drug resistance

Supp. Table 2: Detected microorganisms and corresponding antibiotic resistance profiles in the multi-drug resistant (MDR) group from appendiceal swab samples

**Microorganism**	**Resistant antibiotics**
*Pseudomonas aeruginosa (*3MRGN)*, Klebsiella pneumoniae, Enterococcus avium, Bacterroides ovatus*	Ampicillin, ampicillin/sulbactam, piperacillin/tazobactam, cefotaxime, meropenem
Escherichia coli (ESBL-R/3MRGN)	Ampicillin, ampicillin/sulbactam, cefotaxime, ciprofloxacin, gentamicin
Proteus vulgaris, Escherichia coli (ESBL-R/3MRGN), *Bacteroides thetaiotaomicron, Enterococcus raffinosus*	Ampicillin, ampicillin/sulbactam, cefotaxime, ciprofloxacin, imipenem
Escherichia coli, Bordetella hinzii (3MRGN), *Entecoccus faecium, Streptococcus anginosus, Bacteroides thetaiotaomicron*	Piperacillin/tazobactam, cefepime, levofloxacin, tobramycin
Pseudomonas aeruginosa, Escherichia coli(/ESBL-R), Bacteroides fragilis	Ampicillin, ampicillin/sulbactam, ESBL-R, cotrimoxazole
Escherichia coli (ESBL-R), Enterococcus faecium, Bacteroides thetaiotaomicron	ESBL-R, ampicillin/sulbactam, cefotaxime, imipenem
Klebsiella pneumoniae, Escherichia coli, Acinetobacter baumanii, Bacteroides fragilis	Ampicillin, piperacillin/tazobactam, cefotaxime, fosfomycin
Escherichia coli, Enterococcus faecium, Bacteroides fragilis	Ampicillin, ampicillin/sulbactam, imipeneme
Escherichia coli	Ampicillin, ampicillin/sulbactam, cotrimoxazole, ciprofloxacin
Escherichia coli, Staphylococcus aureus, Strenotrophomonas parasanguinis, Bacteroides ovatus	Ampicillin, piperacillin/tazobactam, clindamycin, levofloxacin
Escherichia coli, Bacteroides thetaiotaomicron	Ampicillin, ampicillin/sulbactam, ciprofloxacin, levofloxacin
Escherichia coli, Klebsiella pneumoniae, Candida albicans, Bacteriodes distasonis, Clostridium innocuum	Ampicillin, ampicillin/sulbactam, piperacillin/tazobactam, clindamycin
Enterobacter cloacae, Bacteroides caccae, Bacteroides distanosis	Ampicillin, ampicillin/sulbactam, cefuroxime
Escherichia coli, Streptococcus anginosus, Staphylococcus lugdunensis	Penicillin, oxacillin, cefuroxime
Citrobacter braakii	Ampicillin, ampicillin/sulbactam, cefuroxime
Escherichia coli, Bacteroides ovatus	Ampicillin, ampicillin/sulbactam
Escherichia coli, Bacteroides ovatus	Ampicillin, ampicillin/sulbactam, cotrimoxazole, gentamicin
Escherichia coli, Acinetobacter calcoaceticus, Parabacteroides distasonis	Ampicillin, ampicillin/sulbactam, piperacillin/tazobactam, clindamycin
Escherichia coli, Enterococcus faecium	Ampicillin, ampicillin/sulbactam, piperacillin/tazobactam, cefuroxime
Staphylococcus aureus	Penicillin G, cotrimoxazole
Escherichia coli, Enterococcus faecium, Bacillus cereus, Bacteroides distasonis	Ampicillin, ampicillin/sulbactam, cotrimoxazole
Serratia marcesscens	Ampicillin, ampicillin/sulbactam, cefuroxime
Escherichia coli, Klebsiella oxytoca, Enterococcus avium, Streptococcus constellatus, Bacteriodes spp.	Ampicillin, clindamycin, cotrimoxazole
Escherichia coli, Pseudomonas aeruginosa	Piperacillin, ciprofloxacin
Enterococcus faecium, Candida glabrata, Candida albicans	Ampicillin, imipenem
Pseudomonas aeruginosa, Escherichia coli, Enterococcus avium, Bacteroides distasonis	Ampicillin, ampicillin/sulbactam, cotrimoxazole
Escherichia coli, Klebsiella oxytoca, Enterococcus faecalis	Ampicillin, fosfomycin
Enterococcus avium, Bacteroides distasonis, fragilis	Penicillin G, clindamycin
Escherichia coli, Enterococcus faecium, Bacteroides thetaiotaomicron, Candida albicans	Penicillin, piperacillin/tazobactam

Each of the 29 patients is represented with their corresponding microbial spectrum and cumulative antibiotic resistance profile. (ESBL-R- Extended spectrum beta-lactamase resistance, 3MRGN- Gram-negative rods that are multi-drug resistant to three of four classes of antibiotics, MDR – multi-drug resistance)
